# Nutrition in the context of the Sustainable Development Goals

**DOI:** 10.1093/eurpub/ckaa034

**Published:** 2020-05-11

**Authors:** Giuseppe Grosso, Alberto Mateo, Natalie Rangelov, Tatjana Buzeti, Christopher Birt

**Affiliations:** c1 Department of Biomedical and Biotechnological Sciences, University of Catania, Catania, Italy; c2 European Programme for Intervention Epidemiology Training (EPIET) Fellow, Istituto Superiore di Sanità, Rome, Italy; c3 Institute of Public Communication (ICP), Faculty of Communication Sciences, Università della Svizzera italiana, Lugano, Switzerland; c4 European Office for Investment for Health and Development, World Health Organization (WHO), Venice, Italy; c5 Department of Public Health and Policy, University of Liverpool, Liverpool, UK

## Abstract

The 2030 Agenda for the Sustainable Development Goals (SDGs) represents a common framework of international cooperation to promote sustainable development. Nutrition is the key point for the SDG 2 ‘End hunger, achieve food security and improved nutrition and promote sustainable agriculture’ and is an essential component for achieving many of the other targets: overall, the nutritional aspects of the SDGs aim to promote healthy and sustainable diets and ensure food security globally. While undernutrition is of minimal concern in the European Union Member States, trends in childhood obesity are still alarming and far from any desirable target. European food production systems have improved over the last years, with immediate impact on several environmental aspects; however, a comprehensive regulatory framework to fulfil the environmental and climate targets is still lacking. Policy actions at multinational level are needed to achieve global nutrition targets designed to guide progress towards tackling all forms of malnutrition while preserving the environment through virtuous food production and food systems.

## Introduction

The global population has increased by nearly 200% in the past 70 years, from 2.6 billion people in 1950 to 7.6 billion in 2017. This increase is projected to continue, reaching a population of nearly 8 billion by 2030 and >9 billion by 2050.^1^ As the average age rises and life expectancy increases over time, the growing number of older people represents a challenge for various systems, including current health and socio-economic systems. Moreover, the rise in global population is of great concern for the environment and it exacerbates the current inability to maintain sustainable development to preserve the needs of future generations. Without preventive actions to promote healthy aging and sustainable development, the risk of chronic diseases and of uncontrolled environmental impacts causing serious threats increases, ultimately resulting in high risk for inequality between generations. In this context, countries around the world adopted the 2030 Agenda for Sustainable Development (United Nations 2030 Agenda) and its 17 Sustainable Development Goals (SDGs). This international policy framework provides a roadmap to end poverty, provide health and prosperity for all while protecting the planet. This review aims to summarize the role of nutrition in the context of the SDGs, focusing on current evidence on sustainable diets and their potential impacts on health and environment.

## The role of nutrition within the SDGs generally

The 2030 Agenda for SDGs represents a common framework of international cooperation to promote sustainable development. To do so, it encourages all countries (low, middle and high income) to reach 17 goals. The SDG 2 (‘End hunger, achieve food security and improved nutrition and promote sustainable agriculture’) is the only SDG that clearly mentions the concept of ‘nutrition’. Despite a reduction of nearly 50 million in the number of children under 5 years of age affected by stunting over the last 20 years, another 150 million are still stunted, 50 million children under 5 years are wasted, and 20 million newborn babies are estimated to be of low birthweight. Although in Europe, these figures are much lower, in the south-eastern European region the prevalence of stunting among children under 5 years may be up to 10%.^2^

It is noteworthy that achieving several SDGs is crucial for achieving the nutrition goal; ‘nutrition’ is an essential component for achieving many of the other SDGs (table 1). Poor nutrition is influenced by several socio-economic factors, including food insecurity attributable to poverty and war (SDG 1). About 800 million individuals globally are suffering from hunger caused by poverty, with 43 million in the European Union (EU) being unable to afford regular quality meals every second day.^3^

**Table 1:** Nutrition in the context of the Sustainable Development Goals

Sustainable Development Goals	Link to nutrition
1. No poverty	Poverty limits access to adequate food intake and makes it difficult to reach nutritional recommendations
2. Zero hunger	Unsustainable food production causes undernourishment
3. Good health and wellbeing	Healthy and sustainable nutrition may reduce premature death including from non-communicable diseases
4. Quality education	Malnutrition affects learning abilities, while higher awareness may affect healthy and sustainable food choices
5. Gender equality	Empowering women to claim their rights leads to improved quality of life and nutrition; proper nutrition improves learning performance, which can be translated into better job opportunities
6. Clean water and sanitation	Access to safe drinking water and sanitation may reduce undernutrition
7. Affordable and clean energy	Creating independence from fossil fuels will reduce greenhouse gas emissions and environmental pollution, and ensure food security
8. Decent work and economic growth	Economic transformation may provide increased nutrition security and sustainable agriculture
9. Industry, innovation and infrastructure	Affordable access to technologies and infrastructure is essential for agriculture development and food security
10. Reduced inequalities	Inequalities cause disparities in income, food, health and education access
11. Sustainable cities and communities	Expansion into rural area increases food needs, creates competition for food and water resources, and finally dependence on food purchases
12. Responsible consumption and production	Meeting the nutritional needs of a growing global population requires sustainable solutions for food production and access to water, as uncontrolled and inefficient food production causes greenhouse gas emissions and soil degradation
13. Climate action	Climate change affects global food production and food security as well as access to fresh water resources
14. Life below water	Aquaculture reduces hunger and improves nutrition; however, overfishing limits biodiversity
15. Life on land	Change of land use causes soil degradation while reducing biodiversity and food production, and decrease access to fresh water
16. Peace and justice	War causes malnutrition and death due to inadequate/insecure food supplies and reduced access to food
17. Partnerships for goals	In order to achieve the goals partnership between both diverse sectors and governments is needed

Education, decent work and economic growth (SDG 4 and 8) have also been associated with better diet quality, although causal inference is still to be explained: preferential consumption of low-quality foods (energy-dense and nutrient-poor) by lower socio-educational classes may be driven by poorer accessibility to, and/or non-affordability of, higher quality foods, among other factors.^4^

Dietary choices are also essential to maintain good health and wellbeing (SDG 3): the so-called double burden of malnutrition is demonstrated, both by nutritional deficiencies (related to higher risk of infectious diseases) and overweight/obesity (associated with increased risk for non-communicable diseases), as a pandemic of gigantic proportions affecting a total of 4 billion people living in both developing and developed countries.^5^ With specific reference to Europe, a north-south gradient of overweight is evident, with a steady rise from 1990 in children and adolescents and alarming prevalence in Greece, Italy, Malta, and Spain, where approximately one in five boys (ranging from 18% to 21%) are obese, while Denmark, France, Ireland, Latvia and Norway are among the countries with the lowest rates (ranging from 5% to 9%).^5^ Furthermore, a healthy body weight is associated with decreased risk for mental health issues, such as low self-esteem, poorer cognitive skills, as well as lower school and work achievements. Besides the aforementioned goals, under- and over-nutrition have been related to rural and urban environments (SDG 11): contrary to the dominant paradigm, more than half of the global rise in mean body mass index (BMI) in the last 30 years is attributable to increases in BMI in rural areas, underlying the importance for planning healthy environments surrounding all housing developments.^6^ Moreover, gender equality (SDG 5) and reduced inequalities (SDG 10) are also related to nutrition. Indeed, particularly in low-income countries, women and girls are essential to agricultural labour; they are also often the ones in charge of preparing food and of taking care of the health of their families and communities. With their knowledge and skills, they can contribute to improved nutrition and to healthier societies.^7^ Food production and consumption have major impacts on environment-related targets (SDGs 6, 7, 9, 12, 13, 14 and 15). Food production is responsible for substantial global greenhouse gas emissions (GHGEs), and release of nitrogen and phosphorus is associated with fertilizer use; food production is also responsible for excessive use of both fresh water and farmland, and for biodiversity loss.^8^ Food consumption also affects environment-related SDGs, as about 20% of food produced in the EU, accounting for 158–298 kg per person per year, is lost or wasted.^9^ Food waste itself is responsible for about 15% of the total GHGEs from the entire food supply chain in Europe.^9^ Ultimately, it is clear that achieving the SDGs is a global challenge that involves a variety of actors (SDG 17), from policy makers to individual consumers; when considering the environmental sustainability and health consequences of food production and consumption, a global food system transformation is likely to be necessary in order to achieve such substantial and ambitious changes.

## Perspectives and challenges for change and reform

Several studies and official reports have been published to raise awareness and to provide evidence-based data supportive of moves towards healthier and environmentally sustainable food production and consumption.^10^ A reduction in consumption of animal-derived foods in favour of plant-derived ones, which may include vegetarian dietary patterns (including ovo-, lacto- and pesco-vegetarian) as well as traditional dietary patterns (such as the Mediterranean diet, the ‘Nordic’ diet, the Japanese diet, etc.), have been shown to improve population health while having lower environmental impact.^10^ A general definition of ‘plant-based dietary pattern’, as one composed of fruit and vegetable, whole-grains, nuts and legumes, fits the conclusions found in the literature produced over the last decades. In the absence of complete restriction of all animal-derived products, fish, milk/dairy products and eggs, only mixed benefits and risks have been reported, while excessive consumption of meat products (especially processed meat) shows a rather linear relation to adverse health outcomes.^10^ However, appropriate application of such proposed changes is challenging, as some plant-based food items are rich in saturated fat (e.g. palm or coconut oil) and others contain added sugars (i.e. from corn syrup). At the same time, the vast international market in palm oil seems to result in environmental problems related to forest depletion in South-Eastern Asia and South America. Thus, plant-based food may not be always good at global level from either health or sustainability/environmental viewpoints.

The EU is committed to the 2030 Agenda with the goal to implement the SDGs within its institutional landscape. However, EU countries have as yet made little headway in the use of SDG indicators and targets to assess necessary progress, to define public policy priorities or to monitor progress made over time.^11^ The EU itself has a rather limited shared competence on public health, subject to a considerable amount of member state autonomy. The EU plays a role in nutrition labelling, regulation of the food industry, farm policy and support and in health education:^12^ some examples of policy approaches have been reviewed recently with a view to drafting a report outlining some preliminary lessons and conclusions, but no univocal proposal can be drafted as yet, nor can one single experience be applied at either European or international levels, as most countries aim to integrate SDGs within the context of existing strategies rather than by creating *ad hoc* programmes. In November 2017, Eurostat released an evaluation report on the EU’s progress towards SDGs based on around 100 indicators, showing criticisms and policy gap analysis requested by the European institutions.^13^ Specifically, the study reported favourable trends—within the limits of the indicators available—for the ‘zero hunger’ SDG 2 in the EU context, focusing on malnutrition as well as on sustainability of agricultural production and its environmental impact: over the last 5 years, percentages of both obese and overweight people in populations have declined, labour productivity in the EU’s agricultural sector has improved, organic farming has grown steadily, public investments in agricultural research and development have increased and nitrate concentrations in groundwater have fallen; on the downside, farmland bird populations continued to decline, and ammonia emissions from agriculture have increased.^13^ The limitations of this report include lack of quantitative thresholds for SDG achievement and disaggregated results for each member state. However, as part of the Common Agricultural Policy (CAP), meat and dairy production (a major cause of GHGEs) still receives massive subsidies within the whole farm subsidy arrangements, but within the current stage of CAP reform, in the light of demands from several non-governmental organizations (NGOs) that this should be replaced by a Common Food Policy, Ursula von der Leyen, the new European Commission President, in her ‘agenda for Europe’ to Parliament, announced that, within her first 100 days, the Commission is shortly to publish an EU consultation on sustainable food policy.^14^

Some reports have been recently published evaluating a variety of criteria for meeting the SDGs (table 2). Two documents from WHO (the World Health Statistics report 2018)^15^ and the World Bank (the Atlas of SDGs),^16^ respectively, provide further insights on SDG achievements at global level, with poor differentiation across countries, with estimates separated by income level, confirming a slowing down of the rising trends of overweight and obesity prevalence in children over the last few years in high-income countries. The latest study from the Global Burden of Disease study group measured the national-level progress on up to 41 health-related SDG indicators used to develop the SDG index from 1990 to 2017, and calculated the rates of change required to meet defined SDG targets at the global level from 2015 to 2030.^17^ In general, the EU member states scored between 59.4 and >74.5 SDG index deciles (with higher scores for Norway, Sweden, UK, Netherlands and Finland). Regarding the SDG 2, while most of them scored optimally on ‘child stunting’ and ‘child wasting’, the study showed significant challenges in reaching acceptable levels of the ‘child overweight’ indicator (all EU countries scoring <40.8 index deciles); other critical indicators in the EU context were ‘alcohol use’ and others not related to nutrition (‘suicide mortality’ and ‘smoking prevalence’). When considering the required global annualized rate of change required to meet each target using the global average in 2015 and specific thresholds to be met by 2030, only a small minority of countries was able to achieve a reduction in child overweight, despite results are still far from the ideal targets (achieved reduction of about 2% compared to the ideal target of 23%). An updated version of ‘The Sustainable Development Report’ prepared by teams of independent experts at the Sustainable Development Solutions Network and the Bertelsmann Stiftung, showed improvements in the general Global Index Score in other EU countries, such as France, Germany, Austria and Czech Republic, despite significant challenges regarding the SDG 2 still remaining, especially in Spain, Portugal, the Netherlands and Poland, while contrasting data have been presented for Norway.^18^

**Table 2:** Indicators used in current reports investigating progression in Sustainable Development Goal 2 ‘Zero hunger’

Report name	Author	Indicators
World Health Statistics 2018: Monitoring health for the SDGs	Global Health Observatory (World Health Organization)	Prevalence of stunting in children under 5 years of age (low height-for-age) Prevalence of wasting in children under 5 years of age Prevalence of overweight in children under 5 years of age
Atlas of Sustainable Development Goals 2018: From World Development Indicators	World Bank	Prevalence of undernourishment Prevalence of stunting in children under 5 years of age (low height-for-age) Prevalence of wasting in children under 5 years of age Prevalence of overweight in children under 5 years of age Extent of food deficit (kilocalories per person per day)
Measuring progress from 1990 to 2017 and projecting attainment to 2030 of the health-related Sustainable Development Goals for 195 countries and territories: a systematic analysis for the Global Burden of Disease Study 2017	Global Burden of Disease study group	Prevalence of stunting in children under 5 years of age (low height-for-age) Prevalence of wasting in children under 5 years of age Prevalence of overweight in children under 5 years of age
Sustainable Development Report 2019	Independent experts	Prevalence of undernourishment Prevalence of stunting in children under 5 years of age (low height-for-age) Prevalence of wasting in children under 5 years of age Prevalence of adult obesity Cereal yield (t/ha) Sustainable Nitrogen Management Index Yield gap closure (%) Human Trophic Level
SDG Progress Digital Report 2019	Food and Agriculture Organization of the United Nations (FAO)	Prevalence of undernourishment Number of people undernourished Prevalence of moderate or severe food insecurity in the population, based on the Food Insecurity Experience Scale Volume of production per labour unit by classes of farming/pastoral/forestry enterprise size Average income of small-scale food producers, by sex and indigenous status (including agricultural output per labour day and average annual income from agriculture) Proportion of agricultural area under productive and sustainable agriculture Number of accessions of plant genetic resources secured in conservation facilities under medium- or long-term conditions Number of animal genetic resources for food and agriculture secured in medium- or long-term conservation facilities Proportion of local breeds classified as being at risk of extinction Agricultural Orientation Index Proportion of countries by region affected by high or moderately high general food prices in the period 2016–17

Recently, a reflection paper entitled ‘Toward a Sustainable Europe by 2030’ published in January 2019 described in general terms the contribution of the EU to the current debate on SDGs at global level, including several aspects related to nutrition^19^: the report emphasizes the need for a modernized CAP, where member states’ national plans will have to reflect strong sustainability principles embedded in the SDGs by adopting a food system based on circular economy principles. Moreover, there is need for involvement of both EU institutions and member states, as well as of municipalities and citizens, to achieve a healthy and environmentally friendly food production, distribution and consumption.^19^ Policy actions at multinational level are needed to tackle all forms of malnutrition (not just undernutrition) if global nutrition targets are to be achieved; such an approach has been suggested to facilitate the achievement of other SDGs that are also related to nutrition.^20^ For instance, food and nutrition security are at the top of the EU’s long-term development cooperation agenda aiming eradication of poverty, food insecurity and undernutrition. However, promotion of programmes and initiatives designed to sustain a global food and agriculture system in line with SDGs may contribute to the achievement of a number of other targets, e.g. providing equitable social development and environmental sustainability, improving employment and income-earning opportunities, value chain development and market integration and implementing demand-driven agricultural research and innovation.^21^ A recent report from the Food and Agriculture Organization of the United Nations provided a digital report including a variety of indicators (as yet not detailed by country) relevant to decision-makers responsible for integrating the goals and targets of the 2030 Agenda for Sustainable Development into national policies and programmes with the aim of transforming food and agriculture and driving achievement across all the SDGs.^22^

Conversely, a number of challenges involve the practical application of implementing the SDGs at European level: education, income and health inequalities across the EU Member States still exist, with the magnitude of inequalities between socio-economic groups larger in some countries than in others, which may vary as according to welfare state regimes; moreover, there is a lack of any comprehensive regulatory framework in line with international obligations, including regulations designed to achieve social, health and environmental targets.^23^ Research areas that deserve further attention in Europe involve the lack of any univocal and complementary metrics specifically dedicated to measure SDGs as well as any qualitative method for reporting on strategies and implementation mechanisms introduced for achievement of the SDGs.

## Conclusions

While explicit in only one SDG, nutrition is an important factor that crosses all the SDGs, in one way or another. Given the current changes in child overweight and obesity prevalence, efforts to reduce the burden of these at global and EU levels need to be improved by strengthening the implementation of targeted interventions. Healthier nutrition, with the adoption of more plant-based dietary patterns, seems to provide the best chance for a healthier population and a healthier planet. National and global actions should promote production of plant-based foods through environmentally sustainable production systems. Furthermore, national and international advocacy and policy changes are needed to promote the shift towards healthy diets. Evidence-based data are essential to provide a roadmap for global change; however, further evidence from the scientific community is needed to continue to examine the association between diet, environmental and human health, and to provide up to date information. Other aspects, such as education, work conditions, gender inequality, clean water and sanitation are of great importance. These factors are, as presented earlier, strongly related to various aspects of nutrition. Thus improved nutrition would contribute significantly towards the achievement of various SDGs. Regarding policy, work needs to be done at production level to provide easier and healthier choices of food at consumption level. Also, interventions targeting the general population are needed to educate and promote behaviour change towards the adoption of healthy and sustainable diets within daily routines. Health care providers should be involved in promoting and educating populations towards healthier and more sustainable lifestyles.

*Conflicts of interest*: The authors declare they have no conflicts of interest.

**Key points**

- Nutrition represents an important pillar of efforts designed to facilitate achievement of Sustainable Development Goals.
- Food security and adoption of healthy and sustainable diets are necessary to meet the proposed target of quality nutrition for all and environmental preservation.
- European Union Member States score optimally against indicators of undernutrition, while childhood obesity remains an alarming issue.
- Environmental impact has lessened slightly, but a comprehensive regulatory framework needed to fulfil achievement of environmental and climate targets is lacking.
